# Polyhydroxyalkanoates in waste activated sludge enhances anaerobic methane production through improving biochemical methane potential instead of hydrolysis rate

**DOI:** 10.1038/srep19713

**Published:** 2016-01-21

**Authors:** Qilin Wang, Jing Sun, Chang Zhang, Guo-Jun Xie, Xu Zhou, Jin Qian, Guojing Yang, Guangming Zeng, Yiqi Liu, Dongbo Wang

**Affiliations:** 1 School of Automation Science & Engineering, South China University of Technology, Guangdong 510640, China; 2 Advanced Water Management Centre (AWMC), The University of Queensland, QLD 4072, Australia; 3 School of Environmental Science and Engineering, Tongji University, Shanghai 200092, China; 4 College of Environmental Science and Engineering, Hunan University, Changsha 410082, China; 5 Key Laboratory of Environmental Biology and Pollution Control, Hunan University, Ministry of Education, Changsha 410082, China; 6 School of Natural and Applied Sciences, Northwestern Polytechnical University, Xi’an 710129, China

## Abstract

Anaerobic sludge digestion is the main technology for sludge reduction and stabilization prior to sludge disposal. Nevertheless, methane production from anaerobic digestion of waste activated sludge (WAS) is often restricted by the poor biochemical methane potential and slow hydrolysis rate of WAS. This work systematically investigated the effect of PHA levels of WAS on anaerobic methane production, using both experimental and mathematical modeling approaches. Biochemical methane potential tests showed that methane production increased with increased PHA levels in WAS. Model-based analysis suggested that the PHA-based method enhanced methane production by improving biochemical methane potential of WAS, with the highest enhancement being around 40% (from 192 to 274 L CH_4_/kg VS added; VS: volatile solid) when the PHA levels increased from 21 to 143 mg/g VS. In contrast, the hydrolysis rate (approximately 0.10 d^−1^) was not significantly affected by the PHA levels. Economic analysis suggested that the PHA-based method could save $1.2/PE/y (PE: population equivalent) in a typical wastewater treatment plant (WWTP). The PHA-based method can be easily integrated into the current WWTP to enhance methane production, thereby providing a strong support to the on-going paradigm shift in wastewater management from pollutant removal to resource recovery.

Activated sludge processes produce plenty of waste activated sludge (WAS), the treatment and disposal of which require substantial costs^1^,^2^,^3^. Anaerobic digestion has been extensively used for WAS treatment due to its ability to achieve methane production and reduce sludge volume simultaneously. However, anaerobic methane production is often restricted by the poor biochemical methane potential and slow hydrolysis rate of the WAS^4^,^5^,^6^. As a result, plenty of pre-treatment approaches including chemical, mechanical and thermal pre-treatment have been proposed to increase methane production by enhancing hydrolysis rate and/or biochemical methane potential^5^,^6^,^7^,^8^,^9^,^10^,^11^. For example, Park *et al.*^11^ reported that methane production from the microwave-treated WAS was 79% higher than that without pre-treatment. However, these methods are mostly cost intensive because of large chemical and/or energy requirements^10^. Also, all of these approaches only focused on the WAS pre-treatment and little attention has been paid to the WAS characteristic itself.

Polyhydroxyalkanoates (PHA), which are carbon and energy storage materials, can be easily accumulated in heterotrophic organisms in wastewater treatment processes^12^,^13^,^14^,^15^,^16^,^17^. PHA accumulation takes place in the presence of excess carbon source. It has been demonstrated that PHA-rich WAS could be produced from the wastewater treatment plants (WWTPs) through wastewater treatment process adjustment and/or operation optimization^12^,^13^,^14^,^15^,^16^,^17^. For instance, Takabatake *et al.*^17^ showed that up to 30% of PHA (on a dry cell weight basis) could be accumulated in the activated sludge biomass of the four real WWTPs. The increase in the PHA level of WAS would lead to the change of WAS characteristics, which might affect methane production in the subsequent anaerobic digestion. Indeed, Huda *et al.*^18^ recently found that methane production from WAS with PHA at 50 mg/g VS (VS: volatile solid) was 25% higher compared with that at 10 mg/g VS. This PHA-based method opens a new door for enhancing anaerobic methane production. However, only one PHA level (i.e. 50 mg/g VS, 10 mg/g VS was used as control) was investigated in the study of Huda *et al.*^18^. Also, the mechanisms responsible for the enhanced methane production are still unknown.

In this work, the effect of PHA levels (i.e. 21, 82, 114 and 143 mg/g VS) of WAS on methane production in anaerobic digestion was assessed systematically using both experimental and mathematical modeling approaches. Anaerobic methane production from WAS with varying PHA levels was experimentally evaluated by biochemical methane potential tests. A model-based analysis was carried out to explore the mechanisms of the PHA-driven improvement in anaerobic methane production. Economic analysis was performed to assess the economic benefit of the PHA-based method. An economically attractive and environmentally friendly integrated PHA-based anaerobic WAS digestion process was also proposed.

## Results

### Effect of PHA levels on biochemical methane production

**Figure 1:**
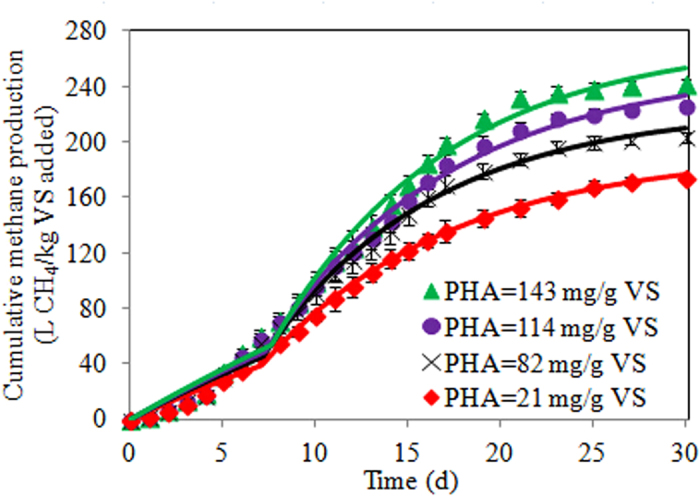
Cumulative methane production from waste activated sludge with varying PHA levels (symbols represent experimental measurements and lines represent model fit). Error bars show standard errors.

Measured methane production from WASs with varying PHA levels throughout the BMP test time is demonstrated in Fig. 1. In general, WASs with higher PHA levels have higher methane production than those with lower PHA levels. For example, the cumulative methane productions were 148, 183, 203 and 225 L CH_4_/g VS added at a digestion time of 20 day when the PHA levels were 21, 82, 114 and 143 mg/g VS, respectively. This reveals that the PHA-based method is capable of enhancing anaerobic methane production.

### Effect of PHA levels on biochemical methane potential and hydrolysis rate

The biochemical methane potential and hydrolysis rate were predicted using a modified first-order kinetic model. The simulated methane production curves are demonstrated in Fig. 1, which suggests that the model can well capture the methane production data (R^2^ > 0.99 in all cases). Table 1 shows the estimated k_1_, k_2_, B_0_, Y and t_lag_ at different PHA levels. In general, there is no significant changes (p > 0.05) in k_1_ (0.03 ± 0.01 d^−1^) and k_2_ (0.10 ± 0.01 d^−1^) in the studied PHA levels (21–143 mg/g VS). This indicates that hydrolysis rate was not significantly affected by the PHA levels. In contrast, B_0_ increased (p < 0.05) with increasing PHA levels, from 192 to 274 L CH_4_/kg VS (increased by 43%) when the PHA levels increased from 21 to 143 mg/g VS. This suggests that PHA-based method could enhance biochemical methane potential of WAS. Correspondingly, the calculated Y increased (p < 0.05) from 0.42 to 0.59 while the PHA levels increased from 21 to 143 mg/g VS, revealing that WAS degradation was improved by 43%. t_lag_ (7 ± 1 d) was similar (p > 0.05) in all PHA levels, indicating that PHA levels applied would not affect lag time.

**Figure 2:**
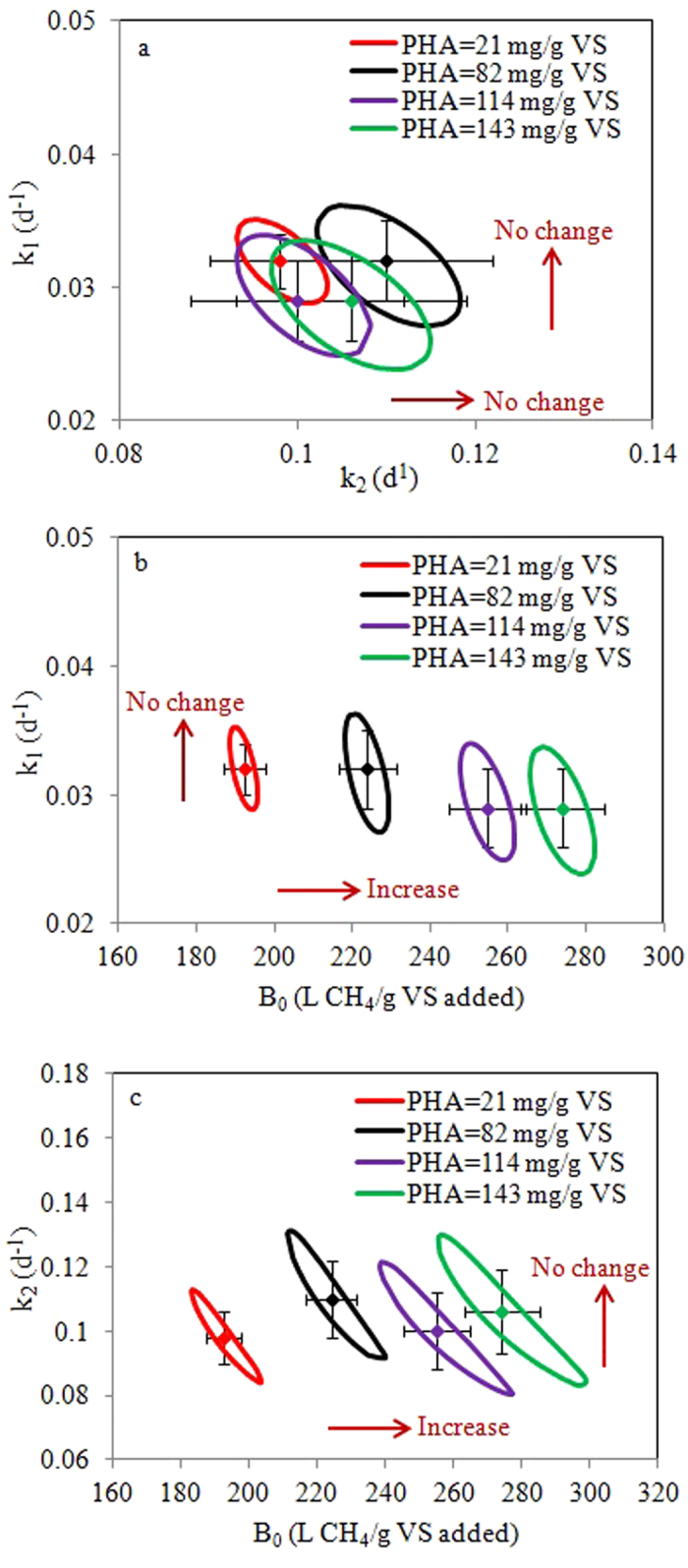
Confidence regions (95%) of the estimated k_1_ and k_2_, k_1_ and B_0_, and k_2_ and B_0_.

95% confidence regions of k_1_ and k_2_, k_1_ and B_0_, and k_2_ and B_0_, were determined to assess their identifiability. The linear confidence intervals (error bars) exceeded the non-linear regions (ellipses) due to the fact that the former was the estimates via four-parameter prediction while the latter was predicted via two-parameter prediction by fixing the other two parameters. The higher degree of freedom and increased localized error function in four-parameter estimation might lead to the over-estimation of the linear confidence intervals^19^. Nevertheless, the overall 95% confidence regions for the three pairs shown in Fig. 2 are small, with mean values lying at the center. This indicates that the parameters are well identifiable and the estimated values are reliable. Figure 2 shows that the 95% confidence regions of k_1_ and k_2_ almost fully overlapped in the studied PHA levels, suggesting that PHA levels would not significantly affect hydrolysis rate. In contrast, the 95% confidence regions of k_1_ and B_0_, and k_2_ and B_0_, moved rightward to the higher B_0_ direction with the increasing PHA levels, revealing that the higher biochemical methane potential can be achieved at the higher PHA levels.

## Discussion

### PHA-based method enhances biochemical methane potential instead of hydrolysis rate

**Figure 3:**
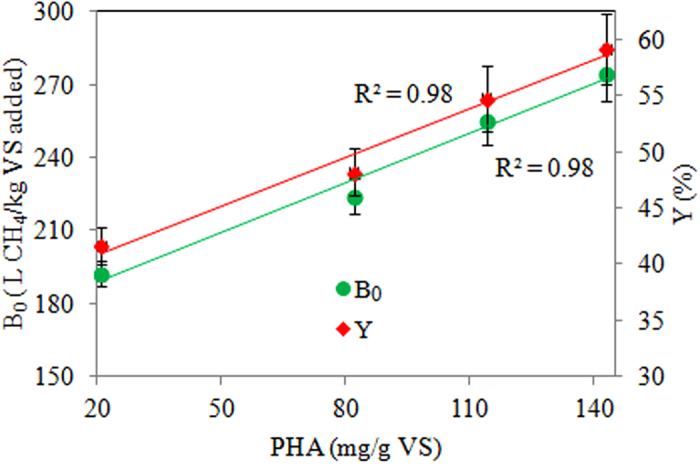
Relationships between PHA levels and B_0_, and PHA levels and Y.

There are two key parameters related to anaerobic methane production, biochemical methane potential (B_0_) and first order hydrolysis rate (k), which represent the extent and speed of anaerobic methane production, respectively. This study showed that the PHA-based method enhances anaerobic methane production through improving B_0_ instead of k. Indeed, a linear relationship between PHA levels and B_0_ was observed (see Fig. 3). This is for the first time that the mechanisms for the PHA improved methane production were revealed. Although Huda *et al.*^18^ demonstrated that the PHA-based method could enhance anaerobic methane production, their study was not systematic and only one PHA level was used. Also, the underlying mechanisms were not revealed in their study. However, it should be highlighted that microbiological analyses will be required in the future to further reveal the mechanisms.

In general, the waste activated sludge (WAS) mainly consisted of protein and carbohydrate. During anaerobic digestion, 1 g of protein (C_4_H_6.1_O_1.2_N, equivalent to 1.53 g of COD) and carbohydrate (C_6_H_10_O_5_, equivalent to 1.18 g of COD) can theoretically produce methane at 0.59 and 0.45 L CH_4_, respectively^20^. In contrast, 1 g of PHA (C_4_H_6_O_2_, equivalent to 1.67 g of COD) can theoretically generate more CH_4_ (0.65 L CH_4_/g PHA) in comparison with protein and carbohydrate^21^. Therefore, if WAS contains more PHA, higher biochemical methane potential would be achieved. This might account for the PHA improved biochemical methane potential of WAS.

The unchanged k revealed in this work indicates that the performance improvement would be impossible or very limited in hydraulically limited anaerobic digesters. However, for anaerobic digesters with a long hydraulic retention time (HRT), the increased B_0_ would drive the enhancement of methane production in the anaerobic digesters.

It has been widely reported that PHA can be accumulated in the main-stream wastewater treatment process by ordinary heterotrophic organisms, polyphosphate-accumulating organisms and glycogen-accumulating organisms^12^,^13^,^14^,^15^,^16^,^17^. The PHA-rich organisms/sludge would then be subject to anaerobic digestion. However, PHA degradation is not taken into account in the current Anaerobic Digestion Model No. 1 (ADM1)^22^. This might lead to an underestimation of methane generation especially for the anaerobic digester receiving PHA-rich sludge. Therefore, the current ADM1 should be modified to include PHA degradation in the future.

### Potential economic benefit of PHA-based method to enhance anaerobic methane production

The results of laboratory BMP tests have been shown to be more conservative or comparable to those of full-scale trials^23^. Consequently, the experimental results attained in this work via the lab-scale BMP tests could be used conservatively for predicting the economic potential of the proposed PHA-based method. This was conducted via a desktop scaling-up study in a WWTP with a population equivalent (PE) of 400,000 and with an anaerobic digester at an HRT of 20 d. Figure 1 shows that WAS with PHA at 143 mg/g VS obtained the largest methane production at a digestion time of 20 d. As a result, this case was chosen for the subsequent economic evaluation. With a 52% increase in methane production at 143 mg PHA/g VS compared with that at 21 mg PHA/g VS (see Fig. 1, 225 versus 148 L CH_4_/kg VS added at a digestion time of 20 d) as demonstrated in this work, the economic benefit is predicted to be approximately $1.2/PE/y compared to the WWTP without PHA-based method (see Table 2). The benefit comes from the improved methane production associated benefit (i.e. its conversion to heat and power) ($0.8/PE/y) and reduced WAS disposal and transport costs ($0.4/PE/y). Therefore, the PHA-based method is potentially economically favorable. Nevertheless, it should be pointed out that the economic analysis results shown here should be regarded indicative only. Particularly, they may change in different regions and/or countries, depending on the local conditions.

### An integrated PHA-based method to enhance anaerobic methane production in wastewater treatment plants

From an integrated economic and environmental perspective, the pollutants (e.g. carbon source) in the wastewater of a WWTP should be managed so that resource recovery from wastewater can be maximized^24^. Based on the findings of this work, an integrated PHA-based method to enhance carbon source recovery in the form of methane in the typical anaerobic/anoxic-aerobic wastewater treatment plants is proposed. The essence of the PHA-based method is to encourage the practitioners to increase PHA content in WAS by process optimization, thereby enhancing anaerobic methane production from WAS.

**Figure 4:**
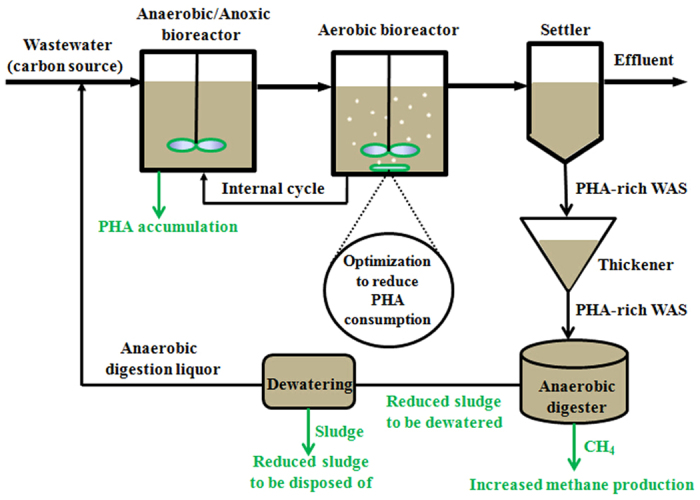
Proposed integrated PHA-based method to enhance methane production in a typical anaerobic/anoxic-aerobic wastewater treatment plant. PHA can be accumulated in the anaerobic/anoxic bioreactor when the carbon source is in excess. Process optimization can reduce PHA consumption in the aerobic bioreactor. The PHA-rich WAS in the anaerobic digester can enhance methane production and reduce sludge production, thereby forming an economically attractive and environmentally friendly method to enable maximized resource recovery in the form of methane. WAS: waste activated sludge.

As shown in Fig. 4, wastewater first entered the anaerobic/anoxic bioreactor, where PHA can be accumulated utilizing the carbon source in the wastewater. In the subsequent aerobic bioreactor, process optimization can be performed to minimize PHA consumption^15^,^16^,^25^. Afterwards, the PHA-rich WAS is fed to the anaerobic digester, in which the enhanced methane production and reduced sludge production can be achieved. This PHA-based method provides a strong support to the on-going paradigm shift in wastewater management from pollutant removal to resource recovery. Also, this method would represent a significant cost reduction in the operation of the WWTPs due to enhanced methane production and reduced sludge production (see previous section). In addition, there is a strong environmental incentive for the PHA-based method, as it avoids external chemical and energy input.

In conclusion, the effect of PHA levels of WAS on anaerobic methane production was evaluated systematically using both experimental and mathematical modeling approaches in this work. It was demonstrated that PHA in WAS enhances anaerobic methane production and higher PHA levels lead to higher methane production in anaerobic digestion. The PHA-based method enhances methane production by improving biochemical methane potential of WAS, whereas the hydrolysis rate of WAS is not affected by the PHA levels. The PHA-based method is potentially environmentally friendly and economically attractive and can be integrated into the current WWTP to enhance methane production, thereby providing a strong support to the on-going paradigm shift in wastewater management from pollutant removal to resource recovery.

## Methods

### Sludge sources

WASs with different PHA levels (defined as WAS-I, WAS-II, WAS-III and WAS-IV, see Table 3) were harvested from four bioreactors with a sludge retention time of 7 day. Seed sludge for the four bioreactors was harvested from a full-scale WWTP in Shanghai, China. All the bioreactors were operated in six hours per cycle, consisting of 240 min aeration, 55 min settling, 5 min decanting and 60 min idle period. These four bioreactors were fed with synthetic wastewaters containing 200, 400, 600 and 800 mg COD/L (COD: chemical oxygen demand), respectively, with acetate as the sole organic carbon source. The other composition of the synthetic wastewaters was described in Wang *et al.*^26^.

The inoculum was collected from a lab-scale anaerobic sludge digester fed with WAS wasted from a full-scale WWTP in Shanghai, China. It will be used in the biochemical methane potential (BMP) tests to be detailed below. The main characteristics of the inoculum were shown in Table 3.

### Anaerobic biochemical methane potential (BMP) tests

BMP tests were used to evaluate methane production from WAS with PHA levels. BMP tests were conducted in 1 L serum bottles (working volume of 600 mL). Every BMP test bottle contained 60 mL inoculum and 540 mL WAS. The bottles were flushed with nitrogen gas for 30 s to remove oxygen. Thereafter, all the serum bottles were capped with rubber stoppers, sealed, and placed in an air-bath shaker at 37 ± 1 °C. Blank containing 60 mL inoculum and 540 mL MilliQ water in the absence of WAS was also set up. pH in all serum bottles was controlled at 7.0 ± 0.1 throughout the digestion period using 4 M HCl or 4 M NaOH with an automatic titrator. All tests were carried out in triplicates. The BMP tests sustained for 30 days, when biogas production decreased to negligible levels. Biogas (CH_4_, H_2_) production was monitored every day in the first two weeks and every 2-3 days thereafter. Biogas production from WAS was calculated by subtracting measured biogas production in an experimental bottle from that measured in the blank bottle. The methane production was recorded as the volume of methane produced divided by the organic dry weight of the WAS added (L CH_4_/kg VS added).

### Model-based analysis

The biochemical methane potential (B_0_) and hydrolysis rate (k), two key parameters relevant to methane production, were adopted to assess and compare methane production potential and kinetics of the WAS with varying PHA levels^4^,^5^. According to the results of BMP tests, a lag period of methane production was observed. Therefore, a modified first-order kinetic model was used to estimate k and B_0_ via fitting the methane production results from BMP tests to the model using a modified version of Aquasim 2.1d^23^,^27^. It should be noted that only methane production results were used to estimate k and B_0_ using the model. This is the commonly used method and is widely used by different groups^28^,^29^,^30^. All parameters were simultaneously predicted using the gradient search method in Aquasim 2.1 d^23^, using the following equations:

When t < t_lag_,


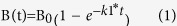


When t > t_lag_,


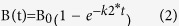


where B(t) = cumulative methane production at time t (L CH_4_/kg VS added); B_0_ = biochemical methane potential (L CH_4_/kg VS added); t = time (d); t_lag_ = lag time of methane production (d); k_1_ = hydrolysis rate during the lag period (d^−1^); k_2_ = hydrolysis rate after the lag period (d^−1^).

The Aquasim 2.1d was also used to estimate the uncertainty surfaces of k_1_, k_2_ and B_0_, based on a model-validity F-test with 95% confidence levels^23^.

The degradation extent (Y) of WAS was determined using the following equation:


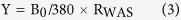


where 380 = theoretical biochemical methane potential of WAS under standard conditions (25 °C, 1 atm) (L CH_4_/kg TCOD)^31^; R_WAS_ = measured ratio of VS to TCOD in the studied WAS.

### Analytical mhethods

The TS and VS levels were determined based on the standard methods^32^. PHA was determined according to the method of Randall and Liu^33^. Protein concentration was measured by the bicinchoninic acid assay method with bovine serum albumin as standard^34^. Carbohydrate concentration was determined by the Anthrone method with glucose as standard^35^. The gas volume was determined through releasing the pressure of the serum bottle using a 300 mL glass syringe to equilibrate with the room pressure based on the method reported previously^36^. The cumulative volume of methane was calculated by the following equation:


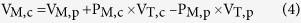


where V_M,c_ and V_M,p_ = cumulative volumes of methane in the current (c) and previous (p) time intervals, respectively. V_T,c_ and V_T,p_ = total gas volumes in the current and previous time intervals, respectively. P_M,c_ and P_M,p_ = percentages of methane measured by gas chromatography in the current and previous time intervals, respectively.

Biogas composition in the collected gas was determined using a gastight syringe with 0.2 mL injection volume and a gas chromatograph (GC112A). The details can be found in Wang *et al.*^26^,^37^.

## Additional Information

**How to cite this article**: Wang, Q. *et al.* Polyhydroxyalkanoates in waste activated sludge enhances anaerobic methane production through improving biochemical methane potential instead of hydrolysis rate. *Sci. Rep.* **6**, 19713; doi: 10.1038/srep19713 (2016).

## Figures and Tables

**Table 1:** Main characteristics of waste activated sludges and inoculum.

Parameter	WAS-I	WAS-II	WAS-III	WAS-IV	Inoculum
TS (g/L)	13.8 ± 0.3	13.9 ± 0.3	13.7 ± 0.4	14.1 ± 0.4	7.2 ± 0.2
VS (g/L)	12.2 ± 0.3	12.5 ± 0.2	12.4 ± 0.3	12.7 ± 0.3	6.3 ± 0.2
PHA (mg/g VS)	21 ± 4	82 ± 8	114 ± 10	143 ± 15	Not determined
Protein (mg/g VS)	594 ± 27	559 ± 29	538 ± 24	510 ± 20	Not determined
Carbohydrate (mg/g VS)	242 ± 16	198 ± 13	177 ± 11	163 ± 14	Not determined

**Table 2:** Estimated k_1_, k_2_, B_0_, Y and t_lag_ at different PHA levels using a modified first-order model (with 95% confidence intervals).

PHA (mg/g VS)	k_1_ (d^−1^)	k_2_ (d^−1^)	B_0_ (L CH_4_/kg VS)	Y	t_lag_ (d)
21	0.03 ± 0.01	0.10 ± 0.01	192 ± 5	0.42 ± 0.02	7 ± 1
82	0.03 ± 0.01	0.11 ± 0.01	224 ± 7	0.48 ± 0.02	7 ± 1
114	0.03 ± 0.01	0.10 ± 0.01	255 ± 10	0.55 ± 0.03	7 ± 1
143	0.03 ± 0.01	0.11 ± 0.01	274 ± 11	0.59 ± 0.03	7 ± 1
Inoculum (mg PHA/g VS)	k_1_ (d^**−1**^)	k_2_ (d^**−1**^)	B_0_ (L CH_4_/kg VS)	Y	t_lag_ (d)
0	0.01 ± 0.01	0.11 ± 0.01	24 ± 1	0.05 ± 0.01	3 ± 1

**Table 3:** Economic analysis of the PHA-based method^a^.

General parameter	Values	
Size of the WWTP (Population equivalent - PE)	400,000	
Size of the WWTP (m^3^ wastewater/d)	100,000	
Influent Chemical Oxygen Demand (COD) (mg/L)	600^b^	
Influent Biochemical Oxygen Demand (BOD) (mg/L)	320^b^	
Influent Total Kjeldahl Nitrogen (TKN) (mg N/L)	55^b^	
Influent ammonium nitrogen (mg N/L)	35^b^	
Influent total phosphate (mg P/L)	8^b^	
Influent total suspended solids (mg/L)	200^b^	
Decay coefficient of heterotrophic biomass (d^**−1**^)	0.2^c^	
Decay coefficient of nitrifying biomass (d^**−1**^)	0.1^c^	
Yield coefficient of heterotrophic biomass (g COD/g COD)	0.625^c^	
Yield coefficient of nitrifying biomass (g COD/g N)	0.24^c^	
Fraction of inert COD generated in biomass decay (g COD/g COD)	0.2^c^	
Sludge retention time in the bioreactor of the WWTP (d)	10^b^	
Mixed liquor suspended solid concentration in the bioreactor (mg/L)	4,000^b^	
Mixed liquor volatile suspended solid concentration in the bioreactor (mg/L)	3,200^b^	
Solids content in thickened sludge	5%^b^	
Solids content in dewatered sludge	15%^b^	
Hydraulic retention time of the anaerobic digester (d)	20^b^	
Methane calorific value (kWh/kg CH_4_)	16^c^	
Conversion efficiency of methane to heat energy	50%^b^,^d^	
Conversion efficiency of methane to power energy	40%^b^,^d^	
Energy price ($/kWh)	0.15^b^	
Cost of sludge transport and disposal ($/wet tonne)	55^b^	
**WWTP without PHA-based method**	Waste activated sludge fed to anaerobic digester (kg TS/PE/y)	8.2
	Waste activated sludge fed to anaerobic digester (kg VS/PE/y)	6.6
	PHA level in waste activated sludge (mg/g VS)	21
	Degradation of secondary sludge in anaerobic digester (on a dry VS basis)	32%^e^
	Remaining sludge after anaerobic digestion (kg TS/PE/y)	6.1
	Remaining sludge after anaerobic digestion (kg VS/PE/y)	4.5
	Methane production (kg CH_4_/PE/y)	0.75
	Energy production from methane (kWh/PE/y)	10.1^f^
	**Benefit due to energy production from methane ($/PE/y)**	**+1.5**^g^
	**Cost of sludge transport and disposal ($/PE/y)**	**–2.2**^h^
**WWTP with PHA-based method**	Waste activated sludge fed to anaerobic digester (kg TS/PE/y)	8.2
	Waste activated sludge fed to anaerobic digester (kg VS/PE/y)	6.6
	PHA level in waste activated sludge (mg/g VS)	143
	Degradation of secondary sludge in anaerobic digester (on a dry VS basis)	49%^e^
	Remaining sludge after anaerobic digestion (kg TS/PE/y)	5.0
	Remaining sludge after anaerobic digestion (kg VS/PE/y)	3.3
	Methane production (kg CH_4_/PE/y)	1.15
	Energy production from methane (kWh/PE/y)	15.5^f^
	**Benefit due to energy production from methane ($/PE/y)**	**+2.3**^g^
	**Cost of sludge transport and disposal ($/PE/y)**	**−1.8**^h^
	**Saving (compared to WWTP without PHA method) ($/PE/y)**	**+1.2**

^a^The calculation methods shown in this table are applicable to any country. However, some parameter values might vary from region to region and from country to country, depending on the local conditions

^b^Personal communication with industry partners.

^c^Refer to Metcalf and Eddy, (2003).

^d^Refer to Carballa *et al.* (2011).

^e^Assumptions based on our results.

^f^Energy production from methane (kWh/PE/y) = Methane production (kg CH_4_/PE/y) × Methane calorific value (kWh/kg CH_4_) × Conversion efficiency of methane to both heat and power energy; Where: Methane calorific value = 16 kWh/kg CH_4_; Conversion efficiency of methane to both heat and power energy = 90%^b,d^. Heat energy can be used to heat up the anaerobic digester and warm the buildings^b^.

^g^Benefit due to energy production from methane ($/PE/y) = Energy production from methane (kWh/PE/y) × Energy price ($/kWh)^b^.

^h^Cost of sludge transport and disposal ($/PE/y) = Remaining sludge after anaerobic digestion (kg TS/PE/y)/Solids content in dewatered sludge × Cost of sludge transport and disposal ($/wet tonne)/1000^b^.
